# Supplementary material for: Resistance to ceftriaxone and penicillin G among contemporary syphilis strains confirmed by natural in vitro mutagenesis
Source: Commun Med (Lond). 2025 Jun 10;5:224. doi: 10.1038/s43856-025-00948-x (PMC12152143; doi:10.1038/s43856-025-00948-x)
Supplement: Supplementary file 1 — Description of Additional Supplementary Files [file 43856_2025_948_MOESM1_ESM.pdf]

DESCRIPTION OF ADDITIONAL SUPPLEMENTARY DATA

File Name: Supplementary Data 1
File Description:

1. **Sheet titled "CTX_data_Figure2+FigureS1"**

The values in this sheet show the *pol*A gene copy number in the *in vitro* culture grown in the corresponding concentration of ceftriaxone (1.25 ng/ml, 2.5 ng/ml, and 5 ng/ml) normalized to *pol*A gene copy number in the control *in vitro* culture grown without ceftriaxone. Values correspond to *in vitro* culture at day 7 and were detected by qPCR. Data for strain SS14 are presented in Figure 2, data for strain DAL-1 are presented in Figure S1.

2. **Sheet titled "PENdata_Figure2+FigureS1"**

The values in this sheet show the *pol*A gene copy number in the *in vitro* culture grown in the corresponding concentration of penicillin (0.125 ng/ml, 0.25 ng/ml and 0.5 ng/ml) normalized to *pol*A gene copy number in the control *in vitro* culture grown without ceftriaxone. Values correspond to *in vitro* culture at day 7 and were detected by qPCR. Data for strain SS14 are presented in Figure 2, data for strain DAL-1 are presented in Figure S1.

3. **Sheet titled "CTXdata_raw_data_FigureS4"**

The values in this sheet show the raw *pol*A gene copy numbers in the *in vitro* culture grown in the corresponding concentration of ceftriaxone (0 ng/ml, 1.25 ng/ml, 2.5 ng/ml, and 5 ng/ml). These values were used to present secondary MIC (minimal inhibitory concentration) in Figure S4 (left half of the Figure S4). Values correspond to *in vitro* culture at day 7 and were detected by qPCR.

4. **Sheet titled "PENdata_raw_data_FigureS4"**

The values in this sheet show the raw *pol*A gene copy numbers in the *in vitro* culture grown in the corresponding concentration of penicillin (0 ng/ml, 0.125 ng/ml, 0.25 ng/ml, and 0.5 ng/ml). These values were used to present secondary MIC (minimal inhibitory concentration) in Figure S4 (right half of the Figure S4). Values correspond to *in vitro* culture at day 7 and were detected by qPCR.
