# Supplementary material for: Resistance to ceftriaxone and penicillin G among contemporary syphilis strains confirmed by natural in vitro mutagenesis
Source: Commun Med (Lond). 2025 Jun 10;5:224. doi: 10.1038/s43856-025-00948-x (PMC12152143; doi:10.1038/s43856-025-00948-x)
Supplement: Supplementary file 2 — Supplemental information [file 43856_2025_948_MOESM2_ESM.pdf]

SUPPLEMENTARY INFORMATION

Petra Pospíšilová, Juraj Bosák, Matěj Hrala, Lenka Krbková, Eliška Vrbová, and David Šmajs

Resistance to ceftriaxone and penicillin G among contemporary syphilis strains confirmed by natural *in vitro* mutagenesis

SUPPLEMENTARY METHODS

Preparation of TP0705 PCR products for natural *in vitro* mutagenesis of *T. pallidum* strains

For *in vitro* mutagenesis, a mixture of five PCR products of different lengths was used. All five PCR products were prepared from a clinical sample with TP0705 allele 1, using a PrimeSTAR GXL DNA polymerase (Takara Bio Europe, Saint Germain en Laye, France). Allele 1 of the TP0705 locus contains two mutations, A1873G and G2122A, compared to allele 10 of the TP0705 locus, which is present in both SS14 and DAL-1 strains. Each 25 µl reaction mixture contained 2 µl of dNTP (200 µM, provided with PrimeSTAR GXL DNA polymerase), 5 µl of 5x GXL buffer, 9.5 pmoles of each primer, 0.5 µl of PrimeSTAR GXL DNA polymerase (0.625 U), and 1 µl of DNA. The PCR touchdown protocol used the following conditions: initial denaturation at 94°C for 1 minute; 8 cycles: 98°C for 10 seconds, 68°C for 15 seconds (annealing temperature gradually reduced by 1°C per each cycle), and 68°C for 16 minutes; 35 cycles: 98°C for 10 seconds, 61°C for 15 seconds, and 68°C for 6 minutes (43 cycles in total); followed by the final extension at 68°C for 7 minutes. The primers and the corresponding PCR product lengths are shown in Table S1.

Table S1. Primers used for PCR amplifications.

Primer sequence	Primer coordinate (Nichols CP004010)*	Product length
5′-AAACCGCGTCAACTACATCC-3′	770247-772266	5604 bp
5′-GAGAAAAGCGGGAGGTAGTG-3′	775831-775850	
5′-CGCGCATACGCAATTTCTGC-3′	771833-771852	3763 bp
5′-AGTACGTGATTTTGCAGCAC-3′	775576-775595	
5′-GCCCTTCCTCCGAGAGAGTA-3′	772296-772315	2888 bp
5′-GTGTGTCTGCCTGAGTGCAT-3′	775164-775183	
5′-GGTCTATATGCAGCCCTTCTTC-3′	772663-772684	1181 bp
5′-GCTTGAGAACGATACCGGATAC-3′	773822-773843	
5′-TGCGGCTTATCCTGATGAATAG-3′	772917-772938	803 bp
5′-TATTCTGCGGCGTTGGATAG-3′	773700-773719	

*Complete genome sequence of TPA Nichols[1]

The PCR products were purified using QIAquick PCR Purification Kit (Qiagen, Valencia, CA, USA) and products were pooled equimolarly with a final concentration of 141 ng per µl.

Monitoring for the presence of mutations at the TP0705 locus

A 200 µl aliqout of treponemal *in vitro* cultures wase used for DNA isolation using QIAamp DNA Mini QIAcube Kit (Qiagen, Hilden, Germany) with an elution volume of 100 µl. Then, 10 µl of eluted DNA was used as a template to amplify the part of the TP0705 locus used for MLST typing.[2] In summary, nested PCR was used to detect the mutagenized region (1st step primers: 5′-GGTCTATATGCAGCCCTTCTTC-3′, 5′-GCTTGAGAACGATACCGGATAC-3′, product size 1181 bp, 2nd step primers: 5′-TGCGGCTTATCCTGATGAATAG-3′, 5′-TATTCTGCGGCGTTGGATAG-3′, product size 803 bp).

The 25 µl reaction mixture for first step contained 2 µl of dNTP (200 µM, provided with PrimeSTAR GXL DNA polymerase), 5 µl of 5x GXL buffer, 0.1 µl of each primer (stock solution 10nM), 0.5 µl of PrimeSTAR GXL DNA polymerase (0.625 U, Takara Bio Europe), water up to 15 µl and 10 µl of DNA. The cycling conditions were: 94°C initial denaturation for 1 minute, 8 cycles of 98°C (10 seconds), 68°C (15 seconds) with touch down protocol of -1 °C per cycle and 68°C (1 minute 45 seconds), followed by 35 cycles of 98°C (10 seconds), 61°C (15 seconds) and 68°C (1 minute 45 seconds), with final extension of 68°C (7 minutes).

The second step was performed with *Taq* polymerase (New England Biolabs, Ipswich, MA, USA). The 25 µl reaction mixture of second step contained 2.5 µl of 10× Standard *Taq* reaction buffer, 0.5 µl of 10mM dNTPs (final conc. 200µM), 0.1 µl of each stock primer solution (100nM), water up to 24 µl, and 1 µl of 1st step PCR product. The PCR conditions were following: 94 °C initial denaturation for 1 minute, 40 cycles of 94 °C (30 seconds), 48 °C (30 seconds), 72 °C (1 minute 45 seconds) and final extension of 72 °C for 7 minutes.

The resulting second-step PCR product was purified using a QIAquick PCR Purification Kit (Qiagen) and sequenced using Sanger sequencing with second-step primers (Eurofins Genomics Europe, Ebersberg, Germany). Sequencing analyses were performed using the Geneious Prime (v 2024.0).

qPCR quantification of treponemal *in vitro* cultures

qPCR detection was based on the detection of the *pol*A gene (TP0105; product size 129 bp) using primers qPCR_polA_F (5'-GAGTGTGCAGTCCGCTATGC-3') and qPCR_polA_R (5'-AGGCAAAAGCGGCATTTCTA-3') and the probe qPCR_polA_probe (5'-FAM-TCCGCTTGGAAACAGCAGGATTG-BHQ-3') as described previously.[3] qPCR reaction was performed in a 20 µl volume containing 10 µl of Luna Universal Probe qPCR Master Mix (New England Biolabs), 0.08 µl of each primer stock solution (final conc. 400 nM), 0.04 µl of FAM-labeled probe stock solution (final conc. 200 nM), 5 µl of template DNA (i.e., sample from treponemal *in vitro* culture), and 4.8 µl of nuclease-free water. qPCR cycling conditions were 95 °C (10 minutes), followed by 40 cycles at 95 °C (10 seconds) and 60 °C (30 seconds). qPCR was performed using an Azure Cielo Real-Time PCR System (Azure Biosystems, Dublin, CA, USA). A standard curve for TPA DNA was constructed using 10-fold serial dilutions (i.e., 10^6 – 10^0 copies/µl) of the pCR2.1-TOPO vector (Invitrogen, Waltham, MA, USA) containing a cloned *pol*A PCR product (129 bp).

SUPPLEMENTARY RESULTS

Fig. S1. Scheme of natural *in vitro* mutagenesis and susceptibility testing of TPA DAL-1 strain to ceftriaxone and penicillin G.

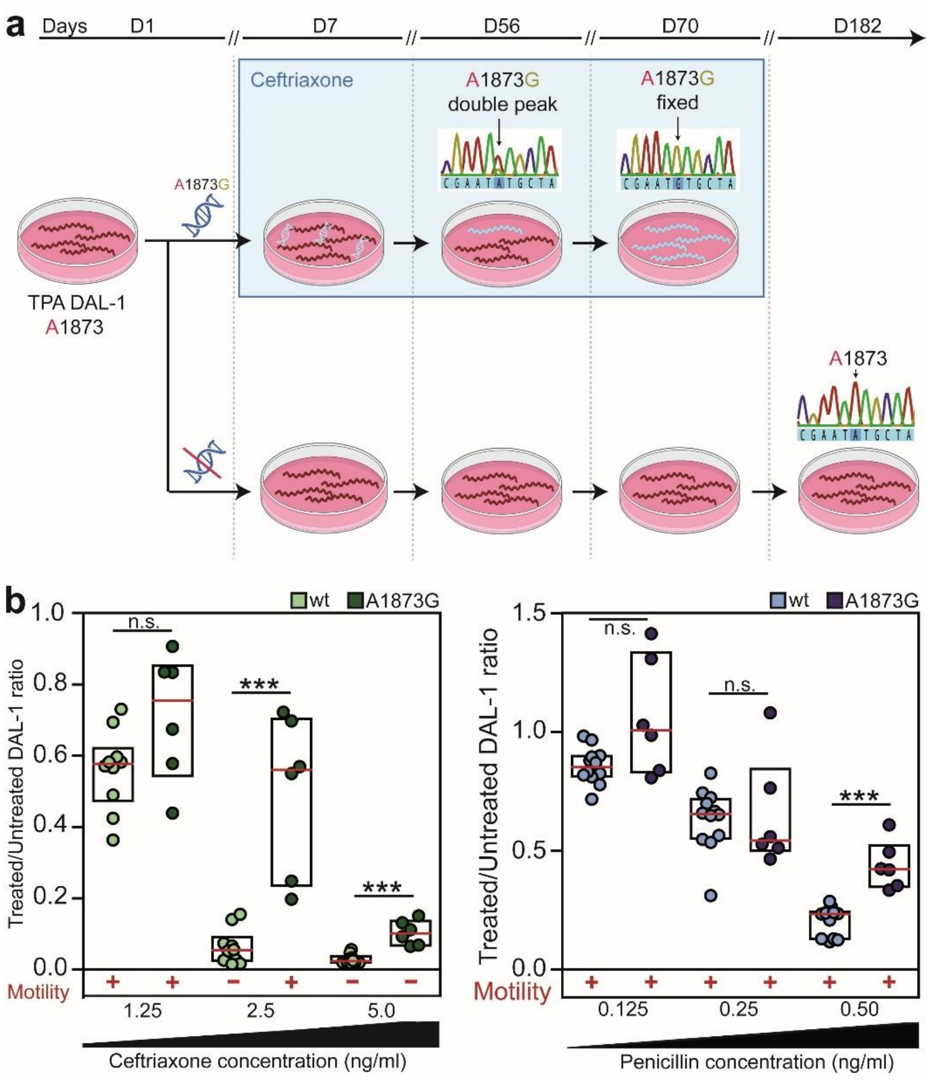

a Scheme of the natural *in vitro* mutagenesis of the TPA DAL-1 strain. The mixture of TP0705 PCR products, containing A1873G and G2122A mutations present in TPA from ceftriaxone failure case, was added to an *in vitro* culture of the DAL-1 strain. From day 7, ceftriaxone (2.5 ng/ml of culture medium) was used for selection of recombinants. At day 70, the A1873G mutation was fixed in the DAL-1 culture. At day 182, this mutation was not detected in the control treponemal cultures without the PCR mixture and ceftriaxone. The treponemal culture without the PCR mixture was eliminated in the ceftriaxone-supplemented control. The arrows indicate position 1873 of the TP0705 gene. The G2122A recombinant was not found during the experiment, so this position is not shown. The scheme contains real sequences from the experiment at relevant time points after mutagenesis. **b** Susceptibility of the recombinant DAL-1 strain to ceftriaxone (left panel) and penicillin G (right panel) determined under *in vitro* conditions. The introduction of the A1873G mutation resulted in decreased susceptibility to both tested antibiotics. The graphs show the numbers of the *pol*A gene detected in *in vitro* culture. All detected *pol*A numbers were normalized to the numbers present in the corresponding controls without antibiotics. The red line represents the median value of the ratio, the boxplot shows the interquartile range, and colored dots represent individual ratio values. At least three biological replicates, each examined twice using qPCR, are shown. Motility data were obtained using dark-field microscopy. The Mann-Whitney test was used to calculate statistical significance (*p < 0.05, **p < 0.01, and ***p < 0.001, n.s. not significant).

Fig. S2. ***Treponema pallidum*** **growth i*n vitro* during selection of recombinant strains SS14 A1873G and DAL-1 A1873G.**

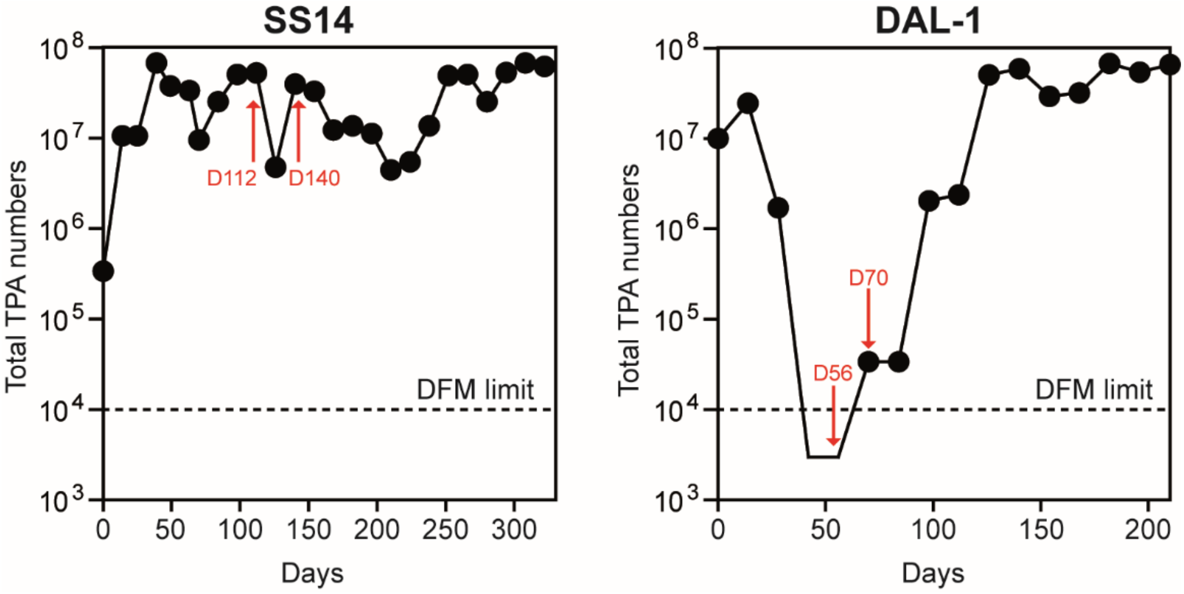

The total numbers of harvested TPA cells during natural transformation of strain SS14 (left) and DAL-1 (right). In the presence of ceftriaxone, TPA populations with A1873G were successfully derived for both strains SS14 and DAL-1. After each 14-day-long subculture period, the total number of TPA cells in *in vitro* culture was quantified microscopically. Simultaneously, the subculture was screened by amplification and sequencing to identify sequence changes in TP0705. The arrows indicate the time points when the A1873G mutation was first detected (double peak) and fixed (single peak) in the TPA culture. Please note the difference in natural transformation between the SS14 and DAL-1 strains. While the SS14 population with the A1873G mutation overgrew the parental population without an eminent decrease in treponemal cells in the *in vitro* culture, the DAL-1 *in vitro* culture experienced a significant drop in treponemal numbers during ceftriaxone selection. Consequently, the mutation was fixed later in the SS14 *in vitro* culture (day 140) compared to the DAL-1 strain (day 70).

Fig. S3. Alphafold structure prediction of TP0705.

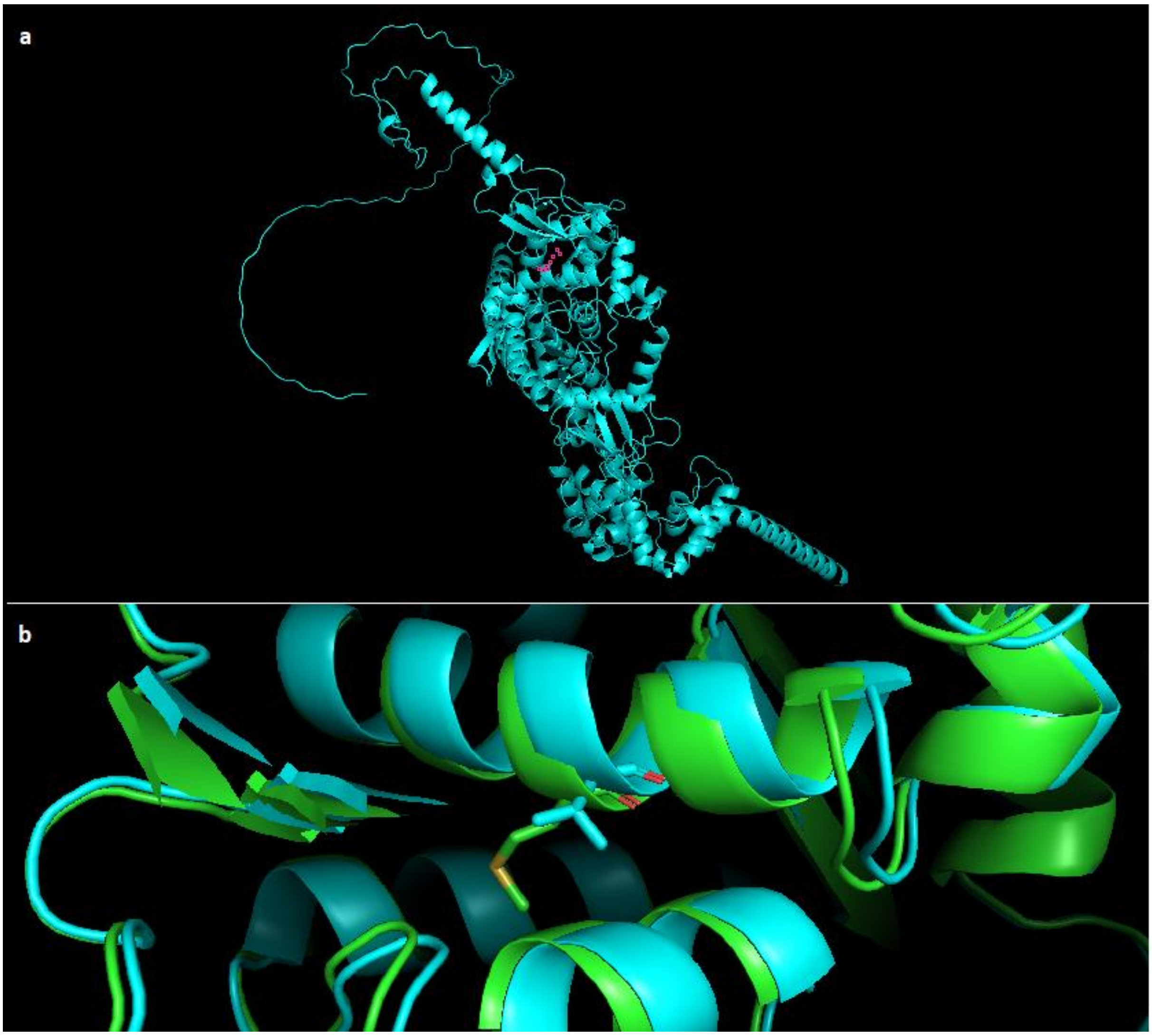

a Structure prediction of wild-type penicillin-binding protein TP0705, with highlighted position 625M (pink), corresponding to the introduction of the A1873G mutation. **b** Detailed structure overlay of wild-type and A1873G-mutated TP0705 (amino acid change M625V). The depicted amino acid change is located within an α-helix and does not significantly alter the overall secondary structure of the protein. The side chain of methionine (M) is shown in green/yellow, and the side chain of valine (V) is shown in blue.

Fig. S4. Susceptibility of TPA to ceftriaxone and penicillin G and determination of minimal inhibition concentration (MIC).

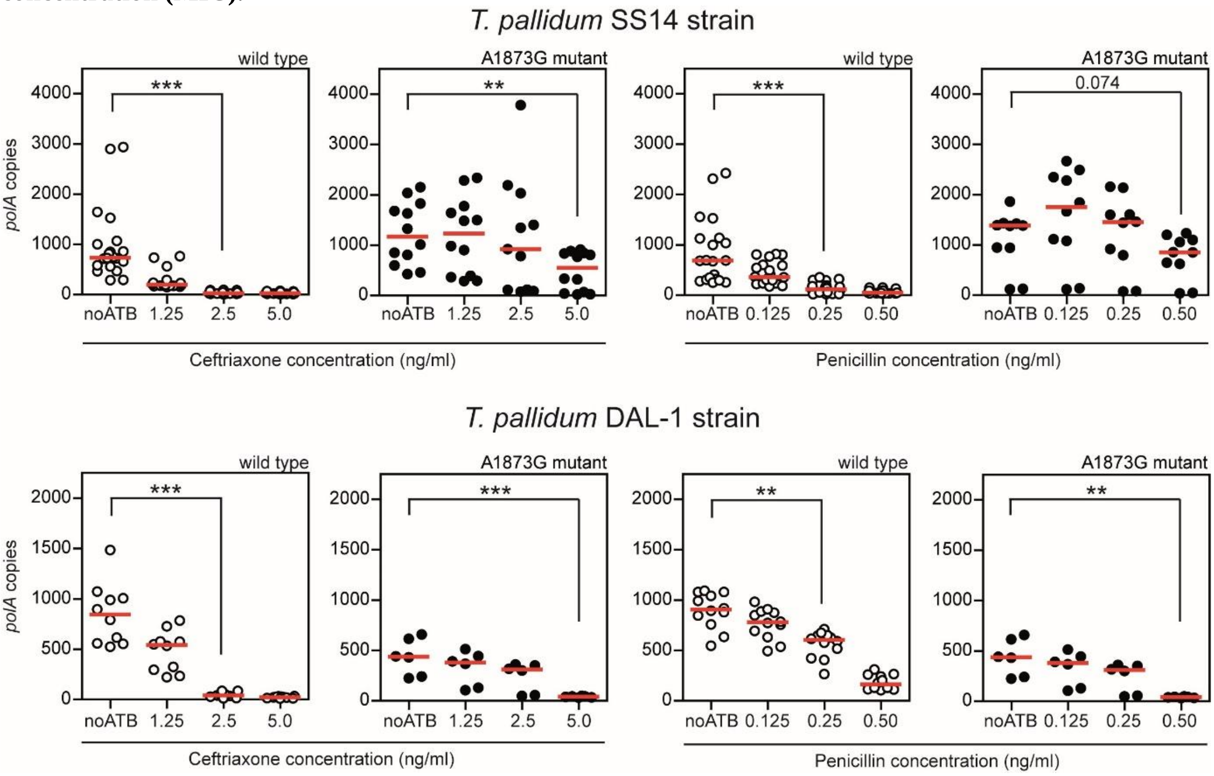

The secondary MIC for SS14 (top) and DAL-1 (bottom) strains and their corresponding recombinant mutants was determined according to Tantalo *et al*.[4] For both recombinant mutants, an increased secondary MIC was observed for ceftriaxone as well as penicillin G was found. The secondary MIC value was defined as the lowest antibiotic dilution where the TPA numbers (i.e., *pol*A qPCR values) were significantly lower than the non-antibiotic control. The resulting secondary MIC values are supported by Kruskal-Wallis mean-rank test, and the p-values shown are based on Dunn´s test (*$p < 0.05$, **$p < 0.01$, and ***$p < 0.001$). The red line represents the median of *pol*A copies from the corresponding number of replicates.

REFERENCES

1. Pětrošová H, Pospíšilová P, Strouhal M, et al. Resequencing of *Treponema pallidum* ssp. *pallidum* strains Nichols and SS14: correction of sequencing errors resulted in increased separation of syphilis treponeme subclusters. PLoS One. 2013;8:e74319.
2. Grillová L, Bawa T, Mikalová L, et al. Molecular characterization of *Treponema pallidum* subsp. *pallidum* in Switzerland and France with a new multilocus sequence typing scheme. PLoS One 2018;13:e0200773.
3. Grillová L, Oppelt J, Mikalová L, et al. Directly Sequenced Genomes of Contemporary Strains of Syphilis Reveal Recombination-Driven Diversity in Genes Encoding Predicted Surface-Exposed Antigens. Front Microbiol 2019;10:1691.
4. Tantalo, L. C., Lieberman, N. A. P., Pérez-Mañá, C., Suñer, C., Vall Mayans, M., Ubals, M., González-Beiras, C., Rodríguez-Gascón, A., Canut, A., González-Candelas, F., et al. (2023). Antimicrobial susceptibility of *Treponema pallidum* subspecies *pallidum*: an in-vitro study. *The Lancet. Microbe*, *4*(12), e994–e1004. https://doi.org/10.1016/S2666-5247(23)00219-7.
