# Supplementary material for: Resistance to ceftriaxone and penicillin G among contemporary syphilis strains confirmed by natural in vitro mutagenesis
Source: Commun Med (Lond). 2025 Jun 10;5:224. doi: 10.1038/s43856-025-00948-x (PMC12152143; doi:10.1038/s43856-025-00948-x)
Supplement: Supplementary file 4 — reporting summary [file 43856_2025_948_MOESM4_ESM.pdf]

nature portfolio

Corresponding author(s): David Šmajs

Last updated by author(s): Apr 16, 2025

Reporting Summary

Nature Portfolio wishes to improve the reproducibility of the work that we publish. This form provides structure for consistency and transparency in reporting. For further information on Nature Portfolio policies, see our Editorial Policies and the Editorial Policy Checklist.

Statistics

For all statistical analyses, confirm that the following items are present in the figure legend, table legend, main text, or Methods section.

n/a	Confirmed	
☐	☒	The exact sample size (*n*) for each experimental group/condition, given as a discrete number and unit of measurement
☐	☒	A statement on whether measurements were taken from distinct samples or whether the same sample was measured repeatedly
☐	☒	The statistical test(s) used AND whether they are one- or two-sided<br>*Only common tests should be described solely by name; describe more complex techniques in the Methods section.*
☒	☐	A description of all covariates tested
☒	☐	A description of any assumptions or corrections, such as tests of normality and adjustment for multiple comparisons
☐	☒	A full description of the statistical parameters including central tendency (e.g. means) or other basic estimates (e.g. regression coefficient) AND variation (e.g. standard deviation) or associated estimates of uncertainty (e.g. confidence intervals)
☒	☐	For null hypothesis testing, the test statistic (e.g. *F*, *t*, *r*) with confidence intervals, effect sizes, degrees of freedom and *P* value noted<br>*Give P values as exact values whenever suitable.*
☒	☐	For Bayesian analysis, information on the choice of priors and Markov chain Monte Carlo settings
☒	☐	For hierarchical and complex designs, identification of the appropriate level for tests and full reporting of outcomes
☒	☐	Estimates of effect sizes (e.g. Cohen's *d*, Pearson's *r*), indicating how they were calculated

Our web collection on statistics for biologists contains articles on many of the points above.

Software and code

Policy information about availability of computer code

Data collection	qPCR data were collected in Azure Cielo Manager,
Data analysis	qPCR data were analyzed in Azure Cielo Manager, GraphPad Prism 10 software was used for statistical analysis, protein structures were downloaded from Alphafold protein structure database or predicted by Alphafold, overlay of protein structures was performed in PyMOL. Quality check of Illumina reads was performed using FastQC (v0.11.5) in conjunction with MultiQC (v1.8). Adapters and low-quality reads were trimmed using Fastp (v0.20.1). Pre-processed reads were mapped against the genome references using the BWA MEM tool (v2.2.1). The overall mapping quality was assessed using QualiMap (v2.2.2), low-quality reads (MAPQ score <60) were filtered out using Samtools (v1.16.1). Variants were identified using Freebayes (v1.3.6).

For manuscripts utilizing custom algorithms or software that are central to the research but not yet described in published literature, software must be made available to editors and reviewers. We strongly encourage code deposition in a community repository (e.g. GitHub). See the Nature Portfolio guidelines for submitting code & software for further information.

March 2021

Data

Policy information about availability of data

All manuscripts must include a data availability statement. This statement should provide the following information, where applicable:

- Accession codes, unique identifiers, or web links for publicly available datasets
- A description of any restrictions on data availability
- For clinical datasets or third party data, please ensure that the statement adheres to our policy

qPCR data data were uploaded into Mendeley data (https://data.mendeley.com/datasets/nkmxcvzc2r/1). Sequencing data were submitted to the NCBI BioProject database under the following accession numbers PRJNA1232603 (SS14 strain carrying A1873G) and PRJNA1232606 (DAL-1 strain carrying A1873G).

Human research participants

Policy information about studies involving human research participants and Sex and Gender in Research.

Reporting on sex and gender	Not applicable
Population characteristics	Not applicable
Recruitment	Not applicable
Ethics oversight	Not applicable

Note that full information on the approval of the study protocol must also be provided in the manuscript.

Field-specific reporting

Please select the one below that is the best fit for your research. If you are not sure, read the appropriate sections before making your selection.

☒ Life sciences ☐ Behavioural & social sciences ☐ Ecological, evolutionary & environmental sciences

For a reference copy of the document with all sections, see nature.com/documents/nr-reporting-summary-flat.pdf

Life sciences study design

All studies must disclose on these points even when the disclosure is negative.

Sample size	At least three biological replicates were performed and analyzed in two technical replicates.
Data exclusions	No data were excluded.
Replication	Samples with the same inoculum were considered to be replicates.
Randomization	All replicates were seeded by the same inoculum and were considered equal so no randomization was necessary.
Blinding	Blinding was not performed

Reporting for specific materials, systems and methods

We require information from authors about some types of materials, experimental systems and methods used in many studies. Here, indicate whether each material, system or method listed is relevant to your study. If you are not sure if a list item applies to your research, read the appropriate section before selecting a response.

Materials & experimental systems

n/a	Involved in the study
☒	☐ Antibodies
☐	☒ Eukaryotic cell lines
☒	☐ Palaeontology and archaeology
☒	☐ Animals and other organisms
☒	☐ Clinical data
☒	☐ Dual use research of concern

Methods

n/a	Involved in the study
☒	☐ ChIP-seq
☒	☐ Flow cytometry
☒	☐ MRI-based neuroimaging

Eukaryotic cell lines

Policy information about cell lines and Sex and Gender in Research

Cell line source(s)	Sf1Ep, skin cells of cottontail rabbit (Sylvilagus floridanus), source: adult male rabbit.
Authentication	The cell line was not authenticated.
Mycoplasma contamination	The cell line was tested regularly for mycoplasma contamination.
Commonly misidentified lines (See ICLAC register)	No commonly misidentified cell lines were used.
